# Collodion for Chordee

**Published:** 1853-08

**Authors:** 


From the Southern Medical and Surgical Journal.

*Collodion for Chordee.*—We read in the "Revue de Therapeutique, &c.," that Dr. Doringer has recently tried the use of Collodion in a case of Chordee, with good effect. The case was that of a young man, who, in his third attack of gonorrhœa, was suffering very much with chordee, which resisted the usual remedies. Dr. D. ordered cold affusions to the organ until relaxation should occur, and then applied a thick coat of collodion to the entire organ. The erections ceased from that moment; but on the next day, the collodion having been removed, they returned, and were again arrested wit another application of the coating.

The above appears to us to be an ingenious and useful application of collodion, but also one not entirely free from danger. If the coating be not carefully applied over the entire organ, including of course the glans, and if it be not of uniform resistance, strangulation might result from the unequal compression whenever there occurred a tendency to erection. The coating should be thick enough to prevent its yielding at any point.

